# P49 Interconnected AMR profiles of *Escherichia coli* in Nigerian live bird markets: evidence from human, poultry, and environmental sampling

**DOI:** 10.1093/jacamr/dlag102.055

**Published:** 2026-06-26

**Authors:** Annelies van Bohemen, Kennedy Chah, Ben Swift, Andrew Mead, Lucy Brunton

**Affiliations:** Pathobiology and Population Sciences, Royal Veterinary College, London, UK; Veterinary Pathology and Microbiology, University of Nigeria, Nsukka, Nigeria; Pathobiology and Population Sciences, Royal Veterinary College, London, UK; Pathobiology and Population Sciences, Royal Veterinary College, London, UK; Pathobiology and Population Sciences, Royal Veterinary College, London, UK

## Abstract

**Background:**

Live bird markets (LBMs) are key points of contact between people, poultry, and the surrounding environment, creating conditions that favour the emergence and transmission of antimicrobial resistance (AMR). *Escherichia coli* is commonly looked to as a One Health AMR indicator organism due to its widespread presence and its capacity to acquire and disseminate resistance genes. Although concern about MDR bacteria is increasing – particularly in low- and middle-income countries – there remains a shortage of data describing resistance patters across the human-animal-environment continuum within LBMs.

**Objectives:**

To characterize phenotypic AMR patterns of *E. coli* isolated from human, poultry, and environmental sources in Nigerian LBMs.

**Methods:**

A cross-sectional study was conducted in four LBMs across Southeast Nigeria between October and December 2024. In total, 487 samples were collected from live-bird sellers (hand swabs, *n*=47), poultry (cloacal swabs, *n*=187), and environmental sites (soil, water, slaughter tables, and cages, *n*=253). *E. coli* isolates (*n*=224) were confirmed using standard biochemical methods. Antimicrobial susceptibility testing against 16 antibiotics spanning 12 antimicrobial classes was performed using the Kirby-Bauer disc diffusion method, with interpretation based on CLSI 2025 guidelines. Differences in resistance proportions across sources were assessed using Fisher’s exact test, and logistic regression was used to estimate odds ratios (ORs) and 95% confidence intervals for MDR, defined as resistance to ≥1 agent in ≥3 antimicrobial classes.

**Results:**

Among the 224 isolates, resistance was highest to tetracycline (85%), ampicillin (80%), and sulfamethoxazole-trimethoprim (68%). The lowest resistance occurred for imipenem (1%), and no resistance to ertapenem was observed. Resistance to ciprofloxacin was observed in 41% of isolates, while resistance to third-generation cephalosporins was rare (<3%). Resistance patterns were highly diverse, with 24% of isolates (54/224) having unique combinations. The median number of antibiotics to which isolates were resistant was 5 (IQR 3–6). MDR prevalence was 83% overall, highest in poultry (85%), followed by the environment (82%) and humans (40%). There was weak evidence against the null hypothesis that there were no differences in MDR prevalence between sources (Fisher’s exact test, *P*=0.055). Logistic regression indicated that human isolates had lower odds of MDR compared with environmental isolates (OR 0.15, 95% CI 0.02–0.96, *P*=0.046), while MDR odds in poultry isolates were similar to those in environmental isolates (OR 1.26, 95% CI 0.61–2.59, *P*=0.53).

**Conclusions:**

The high prevalence of MDR *E. coli* in both poultry and environmental samples underscores LBMs as important reservoirs for AMR at the human-animal-environment interface. Strengthening interdisciplinary One Health surveillance and biosecurity practices in these settings may be critical to mitigating the spread of AMR.

